# Endothelial Cell Permeability during Hantavirus Infection Involves Factor XII-Dependent Increased Activation of the Kallikrein-Kinin System

**DOI:** 10.1371/journal.ppat.1003470

**Published:** 2013-07-18

**Authors:** Shannon L. Taylor, Victoria Wahl-Jensen, Anna Maria Copeland, Peter B. Jahrling, Connie S. Schmaljohn

**Affiliations:** 1 United States Army Medical Research Institute of Infectious Diseases, Fort Detrick, Maryland, United States of America; 2 Integrated Research Facility at Fort Detrick, National Institute of Allergy and Infectious Diseases, National Institutes of Health, Fort Detrick, Maryland, United States of America; Johns Hopkins University - Bloomberg School of Public Health, United States of America

## Abstract

Hemorrhagic fever with renal syndrome (HFRS) and hantavirus pulmonary syndrome (HPS) are diseases caused by hantavirus infections and are characterized by vascular leakage due to alterations of the endothelial barrier. Hantavirus-infected endothelial cells (EC) display no overt cytopathology; consequently, pathogenesis models have focused either on the influx of immune cells and release of cytokines or on increased degradation of the adherens junction protein, vascular endothelial (VE)-cadherin, due to hantavirus-mediated hypersensitization of EC to vascular endothelial growth factor (VEGF). To examine endothelial leakage in a relevant *in vitro* system, we co-cultured endothelial and vascular smooth muscle cells (vSMC) to generate capillary blood vessel-like structures. In contrast to results obtained in monolayers of cultured EC, we found that despite viral replication in both cell types as well as the presence of VEGF, infected *in vitro* vessels neither lost integrity nor displayed evidence of VE-cadherin degradation. Here, we present evidence for a novel mechanism of hantavirus-induced vascular leakage involving activation of the plasma kallikrein-kinin system (KKS). We show that incubation of factor XII (FXII), prekallikrein (PK), and high molecular weight kininogen (HK) plasma proteins with hantavirus-infected EC results in increased cleavage of HK, higher enzymatic activities of FXIIa/kallikrein (KAL) and increased liberation of bradykinin (BK). Measuring cell permeability in real-time using electric cell-substrate impedance sensing (ECIS), we identified dramatic increases in endothelial cell permeability after KKS activation and liberation of BK. Furthermore, the alterations in permeability could be prevented using inhibitors that directly block BK binding, the activity of FXIIa, or the activity of KAL. Lastly, FXII binding and autoactivation is increased on the surface of hantavirus-infected EC. These data are the first to demonstrate KKS activation during hantavirus infection and could have profound implications for treatment of hantavirus infections.

## Author Summary

Primary manifestations of disease due to hantavirus infections include systemic vascular leakage and hypotension for which the underlying mechanism is not known. A particularly perplexing finding is that the vascular endothelium remains intact during hantavirus infection and with no apparent cytopathic effects to explain leakage and edema. Our studies show for the first time that hantavirus-infected EC have increased KKS activation resulting in liberation of the inflammatory peptide, BK. BK is a potent inducer of vascular permeability, edema formation, and hypotension; thus, our results provide a novel mechanism for hantavirus-induced vascular abnormalities. Additionally, we describe the use of an *in vitro* capillary blood vessel model to examine responses occurring locally in blood vessels during infection. This model could be used in future studies by others for assessing further aspects of hantavirus pathogenesis or that of other vascular tropic viruses.

## Introduction

The *Bunyaviridae* family encompasses viruses that cause numerous hemorrhagic fever diseases in humans. The genus *Hantavirus* includes Old World and New World viral lineages. Old World hantaviruses are widespread throughout Asia and Europe and are associated with the clinical syndrome, hemorrhagic fever with renal syndrome (HFRS). The prototype hantavirus, Hantaan virus (HTNV), can cause severe HFRS with a case fatality rate as high as 15% [1], [2]. The New World hantaviruses are the causative agents of hantavirus pulmonary syndrome (HPS) and are found in the Americas [1], [2]. The case fatality rate for HPS is greater than that of HFRS and has been reported to be as high as 50% for Andes virus (ANDV) [1].

While HFRS and HPS differ in the organs exhibiting pathogenic consequences; i.e., kidneys for HFRS and lungs for HPS, both diseases primarily affect blood vessels and cause systemic vascular leakage that can lead to hypotension and shock [1]–[3]. *In vivo* and *in vitro* studies have identified EC as a primary site of viral replication, although hantaviruses can infect epithelial and vascular smooth muscle cells (vSMC) as well [4], [5]. Importantly, despite high levels of viral antigens, the capillary endothelium displays no obvious cytopathology [5], [6]. The mechanism by which hantaviruses cause pronounced vascular leakage when the lining of the endothelium is still intact has remained elusive. It has been assumed that during viral infection, since EC are not damaged, there must be some alteration to the infected cells directly or indirectly through immune mediated processes that result in vascular leakage. One hypothesis implicates the indirect effects of cytokines released from immune cells such as monocytes or T cells. Support for this hypothesis stems from several clinical studies showing that hantavirus-infected patients develop high levels of T cells and cytokine-producing cells, which have been correlated with disease severity (reviewed in [7]). However, in laboratory studies, depletion of T cells was found to have no effect on the outcome of disease in a hamster model that accurately reflects HPS [8].

A second hypothesis centers around observations that vascular endothelial growth factor (VEGF) levels are elevated in HPS and HFRS patients, and that VEGF could affect vascular permeability by increasing the degradation of VE-cadherin [9]–[12]. VE-cadherin is an endothelial specific adhesion molecule that is critical for maintaining cell contact integrity and for regulating vascular permeability [13]. However, in the lethal hamster model of HPS, an upregulation of VEGF was not observed in plasma of ANDV-infected hamsters [14]. In the studies we report here, we developed and tested an alternative hypothesis; i.e., that permeability changes in hantavirus-infected EC are due to increased kallikrein-kinin system (KKS) activation and the liberation of bradykinin (BK).

The KKS consists of serine proteinases; factor XII (FXII) and prekallikrein (PK), and the cofactor, high molecular weight kininogen (HK) [15]. Initiation of this pathway can occur via two mechanisms. The first is dependent upon autoactivation of FXII. FXII can bind to artificial and biological surfaces to induce a conformational change and activation to FXIIa. FXIIa mediates the proteolytic cleavage and activation of PK to kallikrein (KAL) and subsequent cleavage of HK. Alternatively, activation of this cascade can occur independently of FXII. Polycarboxylpeptidase (PRCP) and heat shock protein 90 (HSP90) have been shown to be activators of PK and mediate conversion of PK to KAL and cleavage of HK [16], [17]. However, if FXII is present after initial KAL formation, both enzymes can reciprocally activate causing an amplification of the cascade. In both circumstances the outcome is cleavage of HK resulting in release of a 9 amino acid peptide, BK [18].

BK is an extremely potent inflammatory peptide that exerts numerous effects on the vasculature including vasodilation and increased vascular permeability [19]. BK binds to the bradykinin B_2_ receptor (BKB2R) and triggers an increase in intracellular Ca^2+^, endothelium-derived hyperpolarizing factor (EDHF), prostacyclin (PGI_2_), and nitric oxide (NO) production, eliciting relaxation of vSMC and a drop in blood pressure [20]. Vascular permeability is also increased allowing protein and fluid extravasation across the endothelium via endothelial gaps or trans-endothelial transport [21]. BK has been implicated in the pathogenesis of numerous diseases and disorders including sepsis, pancreatitis, asthma, and most notably the genetic disorder, hereditary angioedema (HAE) [22]–[25]. HAE patients are deficient in C1 esterase inhibitor (C1-INH), an important regulator of activated FXII, which leads to excessive BK generation resulting in edema. Several therapeutics that block BK formation and BK binding have been developed and approved for human use to treat this disorder. The similar disease parameters seen in HAE, HFRS, and HPS led us to examine the importance of BK in the pathogenesis of hantavirus infections. Taken together, our *in* vitro findings of increased KKS activation, clinical data demonstrating activation of PK, and the reported successful treatment of patient with severe HFRS due to Puumala virus infection with a BK antagonist [26] implicates BK in the vascular leakage associated with pathogenic HFRS hantavirus infections.

## Results

### Formation of *in vitro* human capillary blood vessels

**Figure 1:**
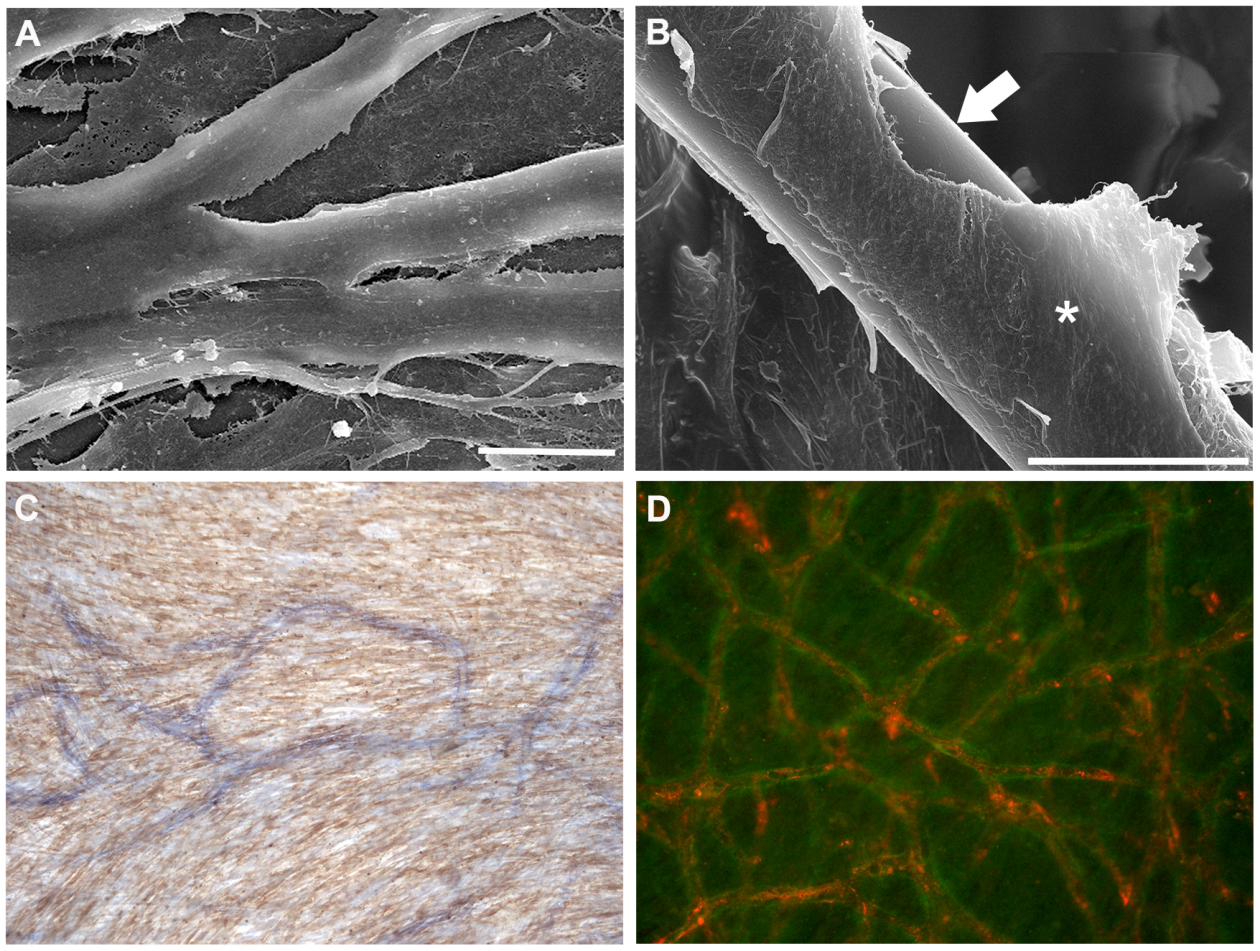
Formation of capillary-like vessels *in vitro*. HUVEC and PaSMC (A & C) or HUVEC and hMSC (B & D) were plated and co-cultured for 5 days to form *in vitro* capillaries. Cells were fixed and processed for scanning electron microscopy. (A) Capillary tubes were long and branched. Size bar = 10 µm. (B) EC tubes (arrow) were stabilized and wrapped by additional cells that were presumably hMSC-differentiated cells (asterick). Size bar = 10 µm. (C) Cells were fixed and stained with antibodies specific for SMA (brown) and COL18A1 (blue) or (D) vWF (red) and COL18A1 (green).

*In vitro* organotypic blood vessel systems are becoming more common for studying the processes associated with angiogenesis. Work by Evenson *et al*. resulted in such a system in which co-culture of human umbilical vein EC (HUVEC) with human mesenchymal stem cells (hMSC) or human pulmonary artery SMC (PaSMC) produced spontaneous formation of capillary-like blood vessels [27]. The capillary-like blood vessels underwent phenotypic changes that are commonly observed *in vivo* such as stabilization of endothelial capillary tubes by PaSMC or hMSC-differentiated cells, formation of adherens junctions, and the presence of basement membrane like structures [27]. We adapted this system as a platform for studying hantavirus pathogenesis. To generate and then verify the formation of blood vessel-like structures *in vitro*, we co-cultured HUVEC with PaSMC or hMSC on silicon chips and processed them for scanning electron microscopy. Upon ultrastructural examination, long and branched EC tubes were visualized throughout the samples (Figure 1A and B). Tubes varied in size with the largest tubes measuring approximately 12 µm in diameter (Figure 1A and B). The tubes were presumably stabilized and wrapped by additional cells that consisted of PaSMC or hMSC-differentiated cells. A matrix morphologically consistent with collagen was observed supporting the capillaries.

To more specifically identify cell types, we fixed and immunostained HUVEC and PaSMC or hMSC co-cultures with antibodies specific for von Willebrand's factor (vWF) or smooth muscle actin (SMA) to identify EC or SMC and collagen XVIII, a vascular basement membrane protein that is normally localized to the peri-endothelial cell region and is indicative of vessel maturity (Figure 1C and D). Numerous EC capillary tubes could be observed in both co-culture samples (Figure 1C and D). Taken together these data demonstrate that we were able to generate an *in vitro* capillary system that could be used as a novel model for studying multicellular involvement during hantavirus pathogenesis.

### Hantaviruses infect *in vitro* capillary blood vessels

**Figure 2:**
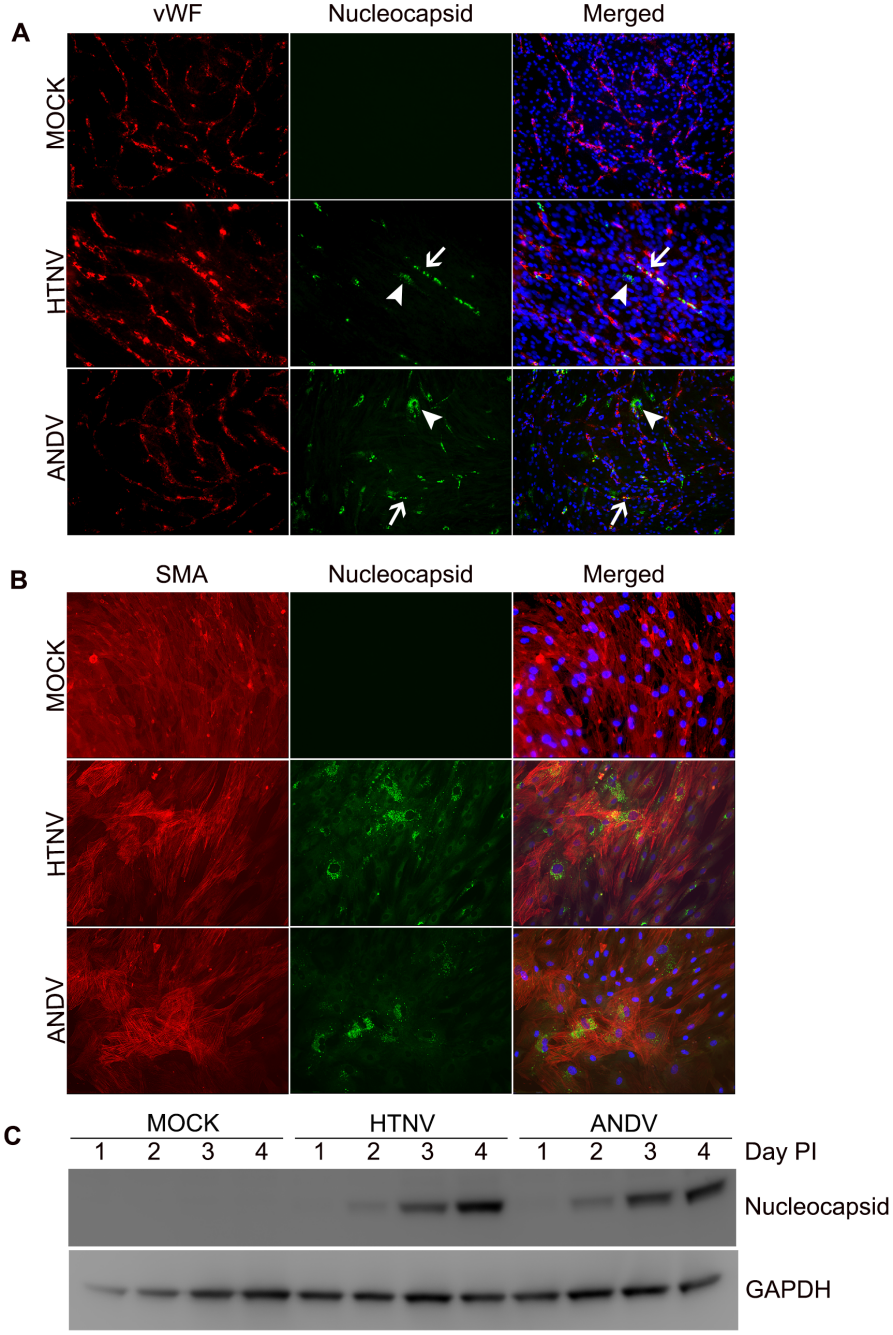
Hantavirus infection of *in vitro* capillaries. HUVEC and PaSMC were plated and co-cultured for 3 days to form *in vitro* capillaries. Samples were then mock-infected or infected with HTNV or ANDV. (A) Cells were fixed and stained with antibodies specific for vWF (red), hantavirus N (green), and nuclei (DAPI). Arrows indicate hantavirus-infected HUVEC and arrowheads indicate hantavirus-infected PaSMC (10×). (B) PaSMC were mock-infected or infected with HTNV or ANDV for 3 days. Cells were fixed and stained with antibodies to SMA (red), hantavirus N (green), and nuclei (DAPI) (20×). (C) PaSMC were mock-infected or infected with HTNV and ANDV for 4 days. Whole cell lysates were harvested on days 1, 2, 3, and 4 and prepared for immunoblotting. Separated proteins were transferred to PVDF membranes and probed with antibodies to detect hantavirus N or GAPDH.

Most *in vitro* studies of hantavirus pathogenesis have been performed by infecting EC alone, and although hantaviruses do predominantly infect this cell type, there are also reports of viral antigen within the perivascular SMC of infected patients [4], [5]. The *in vitro* capillary blood vessel model contains EC and SMC and recapitulates the natural site of infection during hantavirus diseases, so we sought to determine if pathogenic hantaviruses infect both cell types in this model. We found that after infection with HTNV or ANDV, we could detect viral nucleocapsid proteins (N) in the EC capillary tubes (Figure 2A, arrows). Surprisingly, we discovered that HTNV and ANDV antigens were detected in cells not expressing vWF, suggesting that they were also able to infect PaSMC in the capillary blood vessel system (Figure 2A, arrowheads). Similarly, hantavirus N staining patterns were also observed in HUVEC and hMSC co-cultures (data not shown). For subsequent experiments, which compared individual cell types and co-cultures, we used only capillaries formed with HUVEC and PaSMC because PaSMC do not require differentiation.

To determine if vSMC infection was unique to the capillary blood vessel system or if the vSMC alone could be infected, PaSMC were infected with HTNV or ANDV, fixed, and stained with antibodies specific for SMA and hantavirus N. Both HTNV and ANDV N were detected within PaSMC (Figure 2B) indicating that a co-culture system is not required for infection of vSMC. To determine if viral replication was occurring, we also performed a time course infection experiment over 4 days. PaSMC were mock-infected or infected with HTNV or ANDV and whole cell lysates were harvested on 1, 2, 3, and 4 days post-infection and western blots performed to measure the viral N proteins. Increases in N were apparent for both hantaviruses over the 4 days, demonstrating that viral infection and replication in PaSMC were occurring (Figure 2C).

### Pathogenic hantavirus infections do not cause degradation of VE-cadherin in *in vitro* capillary blood vessels

**Figure 3:**
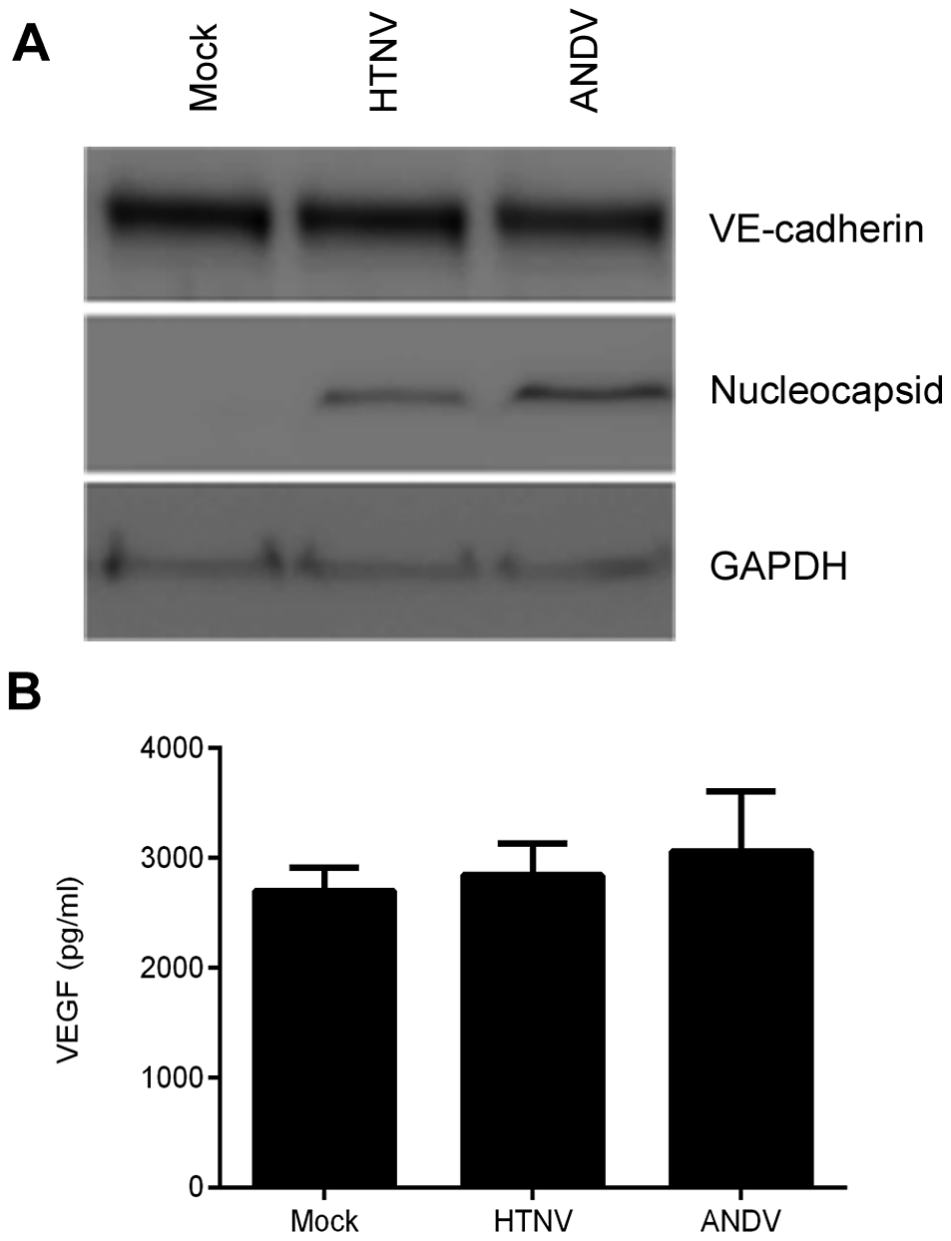
Hantavirus infection of *in vitro* capillaries does not alter VE-cadherin or VEGF levels. HUVEC and PaSMC were plated and co-cultured for 3 days to form *in vitro* capillaries then mock-infected or infected with HTNV or ANDV. (A) Cells were lysed and prepared for immunoblotting. Separated proteins were transferred to a PVDF membrane and probed with antibodies that detect VE-cadherin, hantavirus N, or GAPDH. (B) Supernatants were collected and analyzed by ELISA to measure concentrations of VEGF. Data are represented as mean +/− SEM, n = 4.

VEGF potently mediates vascular permeability through a complex signaling process in which binding to the receptor VEGF-R2 results in loss of the endothelial barrier due to internalization and degradation of VE-cadherin [13]. VEGF has been proposed to mediate hantavirus pathogenesis, as inferred from studies in which adding VEGF to hantavirus-infected HUVEC resulted in hyperphosphorylation of VEGF-R2 and internalization and degradation of VE-cadherin [10]. Another study, however, showed that infection of primary human pulmonary microvascular EC (HMVEC-L) with ANDV alone can direct degradation of VE-cadherin [12]. To further explore this possible mechanism of hantavirus pathogenesis, we tested the effects of HTNV and ANDV infections on VE-cadherin degradation in the *in vitro* capillary blood vessel model. This model should more faithfully represent *in vivo* conditions than EC alone because it contains both cell types normally present in human blood vessels and secretes VEGF [27]. HUVEC and PaSMC were co-cultured to form *in vitro* capillary blood vessels and mock-infected or infected with HTNV or ANDV for 3 days without VEGF-containing media. Whole cell lysates of HTNV- or ANDV-infected samples were examined for the presence of VE-cadherin by western blotting. There was no noticeable degradation of VE-cadherin in the virus-infected samples (Figure 3A), despite the presence of HTNV and ANDV antigens in both HUVEC and PaSMC as determined by IFA. VEGF was detected in all supernatants at 3 days post-infection with no statistically significant levels in hantavirus samples when compared to mock (Figure 3B). In addition, we did not observe abnormalities in vessel structures, which we would expect if VE-cadherin levels were reduced as VE-cadherin is also recognized to be essential for maintaining the structural integrity of newly formed blood vessels [28]. These results demonstrate that infection with pathogenic hantaviruses in a blood vessel model that secretes VEGF does not cause loss of vascular integrity.

### The liberation of bradykinin is increased on hantavirus-infected *in vitro* capillaries, vascular smooth muscle cells, and endothelial cells

**Figure 4:**
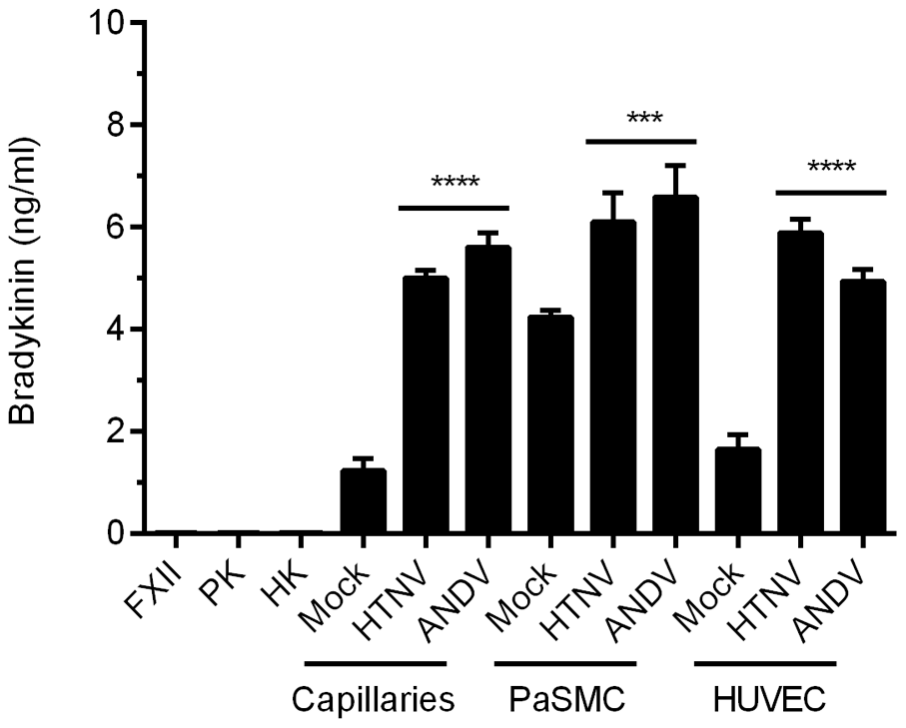
Liberation of bradykinin on hantavirus-infected *in vitro* capillaries, PaSMC, and HUVEC. Capillaries, PaSMC, and HUVEC were plated separately and mock-infected or infected with HTNV or ANDV for 3 days. Cells were treated with 1 µM HOE 140 and 5 µM Trandolapril and incubated with 50 nM of FXII, PK, and HK in buffer for 1 h at 37°C. Supernatants were collected and analyzed using a bradykinin enzyme immunoassay. As controls, FXII, PK, and HK were also tested individually for non-specific binding in the immunoassay. Data are represented as mean +/− SEM, n = 6. ***p≤0.001, ****p≤0.0001.

Hantavirus-infected EC would likely be exposed to blood plasma proteins *in vivo*, providing an opportunity for their participation in dysregulating endothelial cell barrier functions through the release of BK, which in turn would immediately affect vascular permeability. To test whether the KKS can result in altered levels of BK, HUVEC and PaSMC were plated and co-cultured to form *in vitro* capillaries and then were mock-infected or infected with HTNV or ANDV. The plasma factors FXII, PK, and HK were added to cultures and BK in the supernatants was measured using an enzyme immunoassay. Significantly higher levels of BK (p≤0.0001) were identified in supernatants from both HTNV- and ANDV-infected capillaries as compared to the mock-infected samples (Figure 4).

To determine if BK elevations occur in individual cell types, PaSMC and HUVEC were also cultured separately and mock-infected or infected with HTNV or ANDV. Plasma components FXII, PK, and HK were added to the cultures, and BK levels in cell supernatants were measured. We detected significantly higher levels of BK in the supernatants of HTNV- (3.5-fold) and ANDV-infected (3-fold) HUVEC as compared to mock-infected samples, and a modest increase in PaSMC (p≤0.001) (Figure 4).

### Increased cleavage of kininogen on the surface of hantavirus-infected endothelial cells

**Figure 5:**
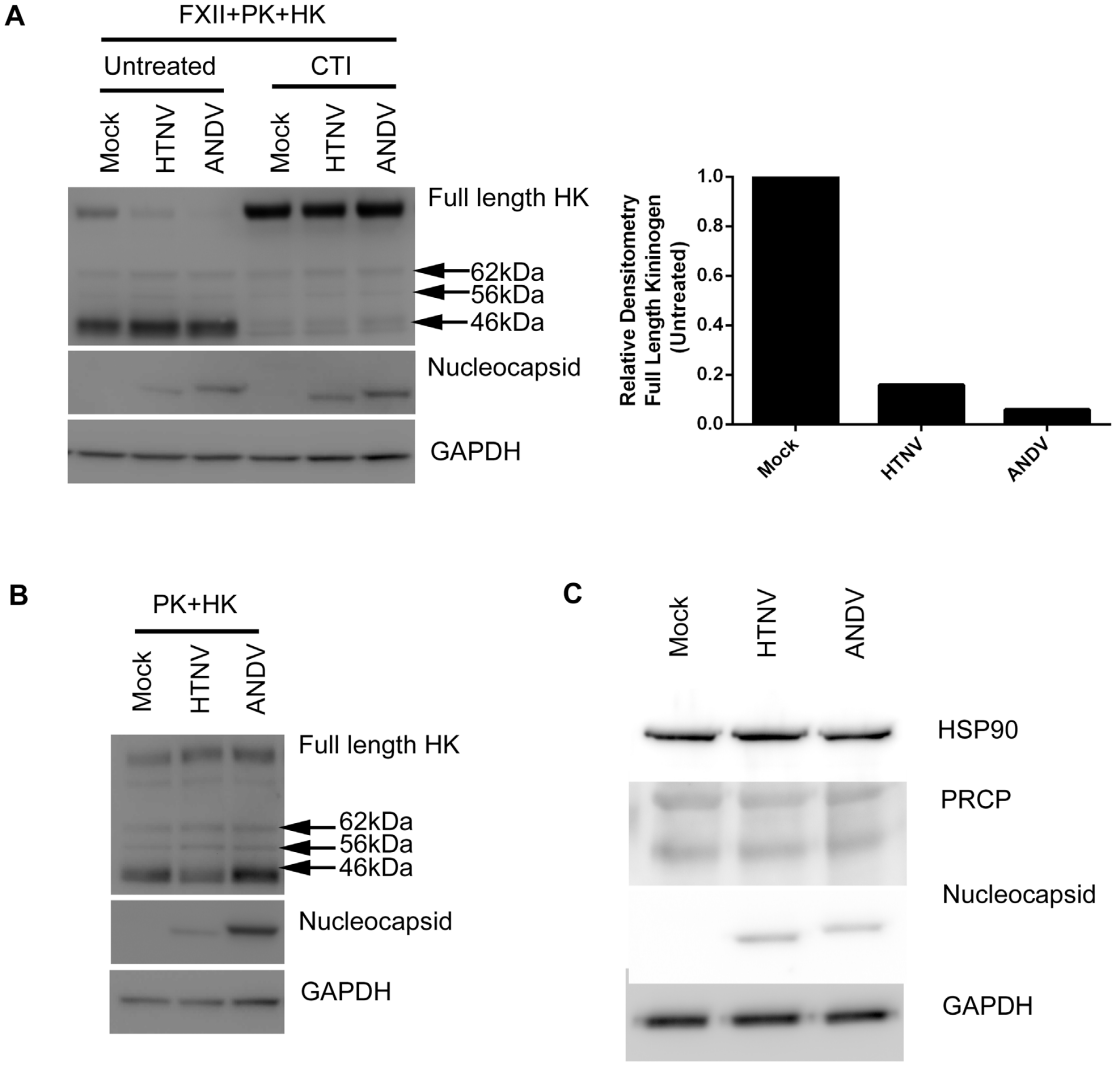
Cleavage of HK on hantavirus-infected HUVEC. HUVEC were plated and mock- infected or infected with HTNV or ANDV for 4 days. 50 nM of HK in buffer was added to the monolayer and incubated for 1 h at 37°C. The cells were incubated an additional 1 h with (A) 50 nM FXII and 50 nM PK in the absence or presence of 1 µM CTI or (B) 50 nM PK. To examine cleavage of HK, cells were lysed and prepared for immunoblotting. Full length, heavy, and light chain fragments of HK, hantavirus N, or GAPDH antibodies were used to detect protein. Levels of full length HK were analyzed by densitometery. (C) Samples were examined by immunoblotting to assess the levels of PRCP and HSP90 in mock-, HTNV-, and ANDV-infected HUVEC.

While KKS activation can occur on the surface of normal uninfected HUVEC, the increased levels of BK that we observed in the supernatants of hantavirus-infected capillaries or individual cultures of PaSMC or HUVEC suggested an increase in cleavage of HK was occurring. FXII can amplify the KKS through reciprocal activation with PK and cleavage of HK yielding BK; however, in the absence of FXII, other proteins such as PRCP and HSP90 can mediate the activation of PK and subsequent cleavage of HK. To determine if FXII was required for increased HK cleavage in hantavirus-infected EC, we mock-infected or infected HUVEC with HTNV or ANDV and incubated with FXII, PK, and HK or only PK and HK. When FXII was present, we observed a significant decrease of full length HK in HTNV- and ANDV-infected samples as compared to the mock and evident by densitometry (Figure 5A). We also observed the appearance of cleavage products, a heavy chain (62 KDa) and two light chain products (56 KDa and 46 KDa) [29] (Figure 5A). Moreover, treatment of the mock- or hantavirus-infected HUVEC with the FXIIa inhibitor, corn trypsin inhibitor (CTI), resulted in increased full length HK in all samples, indicating that FXII was important for this process (Figure 5A). In contrast, when exogenous FXII was not present, similar amounts of full length HK and cleavage products were detected in mock-, HTNV-, and ANDV-infected samples (Figure 5B). Treatment with the KAL inhibitor, PKSI-527, but not CTI was able to inhibit cleavage of full length kininogen indicating that KAL was present in our samples (data not shown). Additionally, when only HK was added, we did not observe a significant difference in the levels of HK on the surface of mock- and hantavirus-infected HUVEC by western blotting, indicating that hantavirus infection by itself does not increase HK binding (data not shown).

Although it has not been clearly established how PRCP or HSP90 activates PK, we considered whether viral infection might increase the levels of these proteins and lead to increased PK activation. To eliminate a role for these proteins in increased PK activation, we tested levels of PRCP and HSP90 in mock-, HTNV-, and ANDV- infected HUVEC and detected no apparent differences by western blot (Figure 5C).

### Plasma factor enzyme activity is increased on the surface of hantavirus-infected endothelial cells

**Figure 6:**
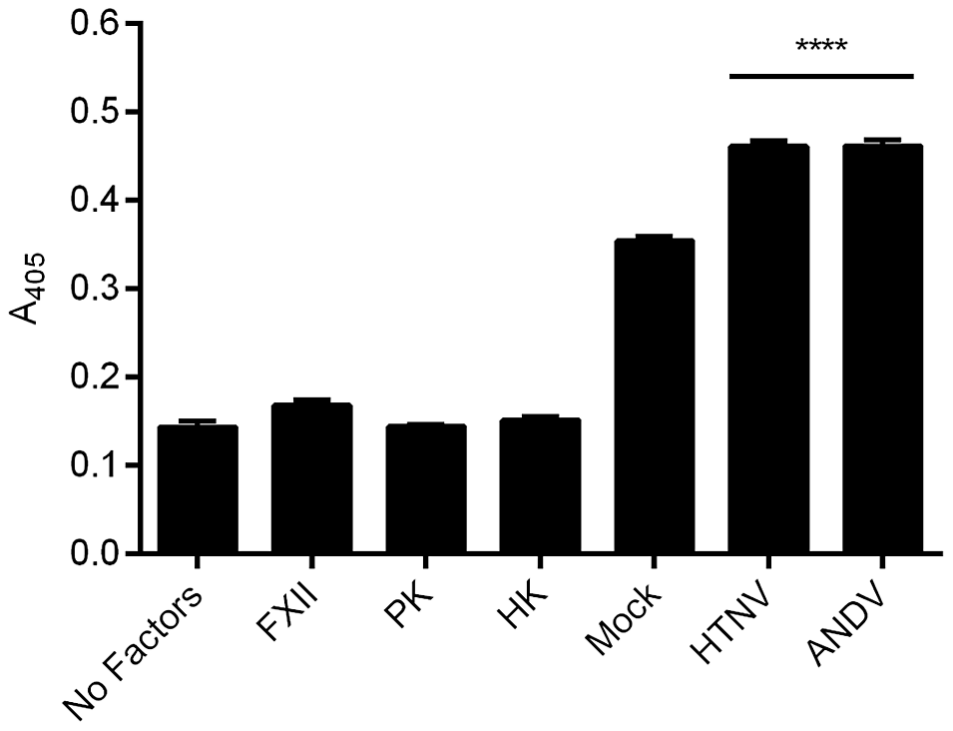
Enzymatic activity of FXIIa and KAL on hantavirus-infected HUVEC. HUVEC cells were plated and mock-infected or infected with HTNV or ANDV for 4 days. 20 nM of HK in buffer was added to the monolayer and incubated for 1 h at 37°C. To measure enzyme activity cells were washed and incubated with 20 nM FXII, 20 nM PK, and 0.8 mM S2302 for 1 h at 37°C. The liberation of paranitroanilide (pNA) from substrate (S2302) was measured at 405 nm. As controls, FXII, PK, and HK were also added individually to wells. Data are represented as mean +/− SEM, n = 12. ****p≤0.0001.

Increased levels of BK and increased cleavage of HK suggest that increased activation of the enzymes FXII and PK is occurring on the surfaces of HTNV- and ANDV-infected HUVEC. To assess enzyme activities, we incubated mock- or hantavirus-infected HUVEC with FXII, PK, and HK and used a chromogenic substrate, S2302, with specificity for the active enzymes FXIIa and KAL. Consistent with our data showing increased cleavage of HK and subsequent release of BK, we also detected significantly higher FXIIa/KAL hydrolytic activity in the presence of HTNV and ANDV when compared to mock-infected samples (Figure 6). In the absence of FXII, no significant difference in KAL activity was detected between mock and hantavirus-infected HUVEC, further supporting the importance of FXII for the increase in KKS activation of hantavirus-infected EC (data not shown).

### Activation of the kallikrein-kinin system alters the permeability of hantavirus-infected endothelial cells

**Figure 7:**
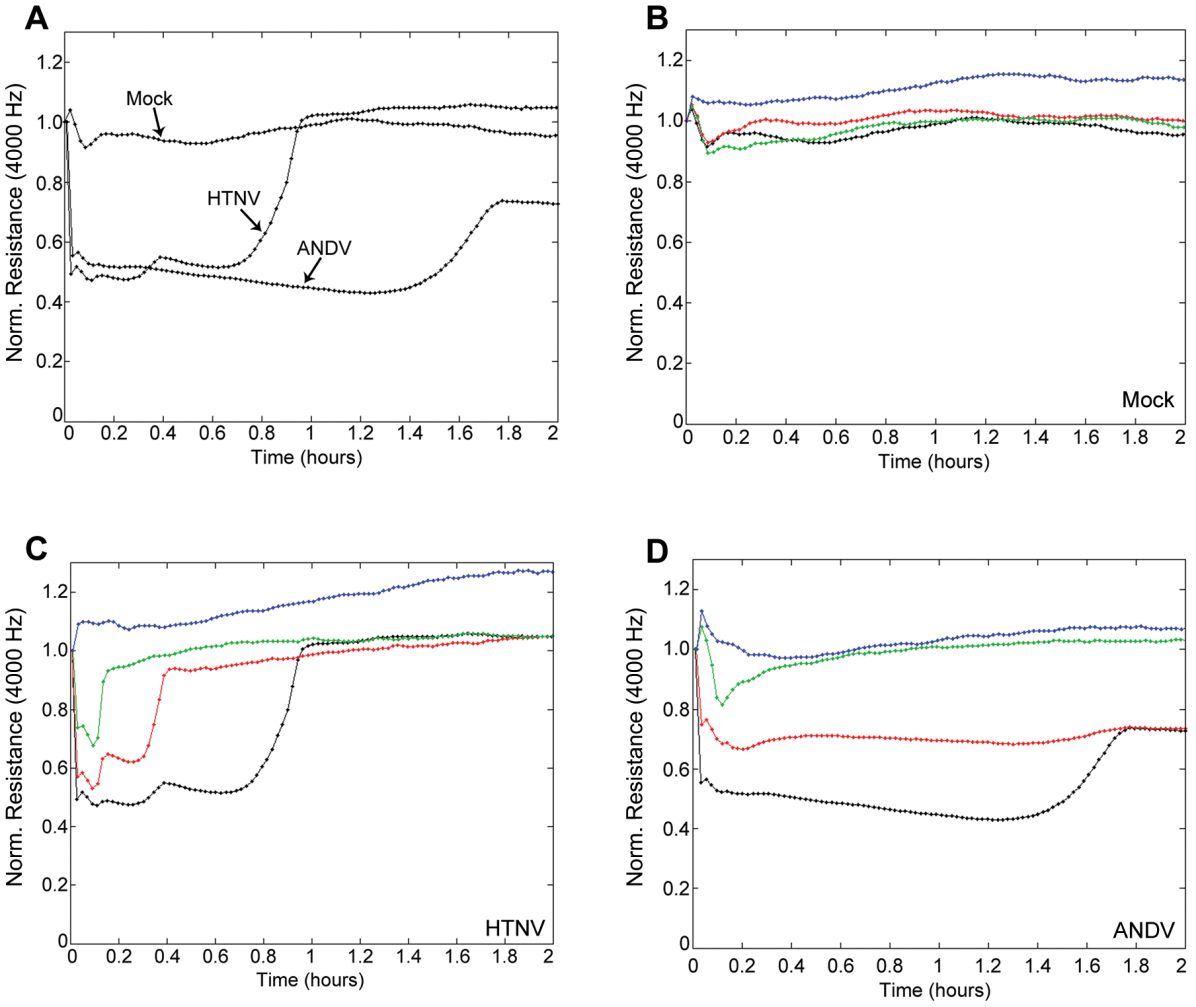
Kallikrein-kinin system activation and changes in permeability of hantavirus-infected HUVEC. Mock-, HTNV-, or ANDV-infected HUVEC were trypsinized, seeded onto ECIS chamberslides, and cultured until confluent. Media were removed and replaced with phenol red free EBM containing Zn^2+^. After an equilibration, 0.2 ml of phenol red free EBM containing Zn^2+^, FXII, PK, and HK (100 nM each) were added to cells (time zero) (black lines) (A). To measure inhibition of activation, some samples were treated with CTI (1 µM) (blue line), PKSI-527 (5 µM) (red line), or HOE 140 (1 µM) (green line) simultaneously with factors (B, C, & D). Real time measurements frequency measurements were taken throughout the assay.

Our data showing that HTNV and ANDV can alter enzyme activation of FXII and PK, cleavage of HK, and liberation of BK, led us to hypothesize that BK would induce profound changes to EC and increase vascular permeability. Generally, changes in permeability are identified using transwell systems, in which leakage of a tracer molecule is measured by passage from the upper chamber to the lower chamber over a course of 1–2 h. However, the biological half-life of BK is extremely short; lasting approximately 15 sec and its effects are rapid [30]. To capture this rapid process, we implemented electric cell-substrate impedance sensing (ECIS), which allows for real-time measurements of resistance and alterations in permeability of cultured cells. Mock- or hantavirus-infected HUVEC were cultured on ECIS arrays and the effects of added FXII, PK, and HK on resistance measurements were continuously recorded throughout the experiment. Time 0 h indicates that point at which plasma factors with or without inhibitors was added to cells already mock-, HTNV-, or ANDV-infected. An initial drop in barrier function was observed, even in mock-infected cells, upon addition of plasma factors FXII, PK, and HK. This is expected as addition of any type of solution to the wells will cause a negligible and transient drop in resistance due to temperature fluctuations, shear stress that is induced when media is added to wells, and effects of the factors themselves. The maximum decrease in resistance that we observed in the mock-infected cells was 10% (Figure 7A). These findings are consistent with previously reported changes on the ECIS after treatment of cells with BK alone [31]. In stark contrast, wells that contained HTNV- or ANDV-infected HUVECs exhibited a profound decrease in barrier function of approximately 50% as compared to the initial resistance measured at 4,000 Hz. The effect occurred at a more rapid rate than observed in the mock-infected wells and was seen immediately upon addition of the factors (Figure 7A).

To further explore the role of FXII, PK, and BK on the reduction in barrier function, we also treated cells with either the FXIIa and KAL inhibitors CTI or PKSI-527, respectively or the BK receptor antagonist, HOE 140 (Figure 7B, C, & D). Addition of these compounds had a marked positive impact on the ability of the endothelium to circumvent virus-directed permeability changes. Indeed, CTI was able to completely protect the endothelial monolayer from decreases in barrier function (Figure 7C & D). These data support and demonstrate functionally the critical role of FXII in the cleavage events leading to permeability changes associated with hantavirus infections. Cells treated with HOE 140 also exhibited a strong protective effect, with cells maintaining a greater percentage of initial barrier function over untreated controls and again exhibiting a more rapid recovery (Figure 7C & D). Finally, addition of PKSI-527 is partially protective as cells treated with this inhibitor exhibited a reduced change in barrier function as compared to untreated cells and they recovered faster as well (Figure 7C & D). Together these data suggest that blocking the activities of FXIIa and the BK receptor itself have the greatest protective effect for maintaining endothelial integrity of hantavirus-infected HUVECs.

### FXII binding and autoactivation is increased on hantavirus-infected endothelial cells

**Figure 8:**
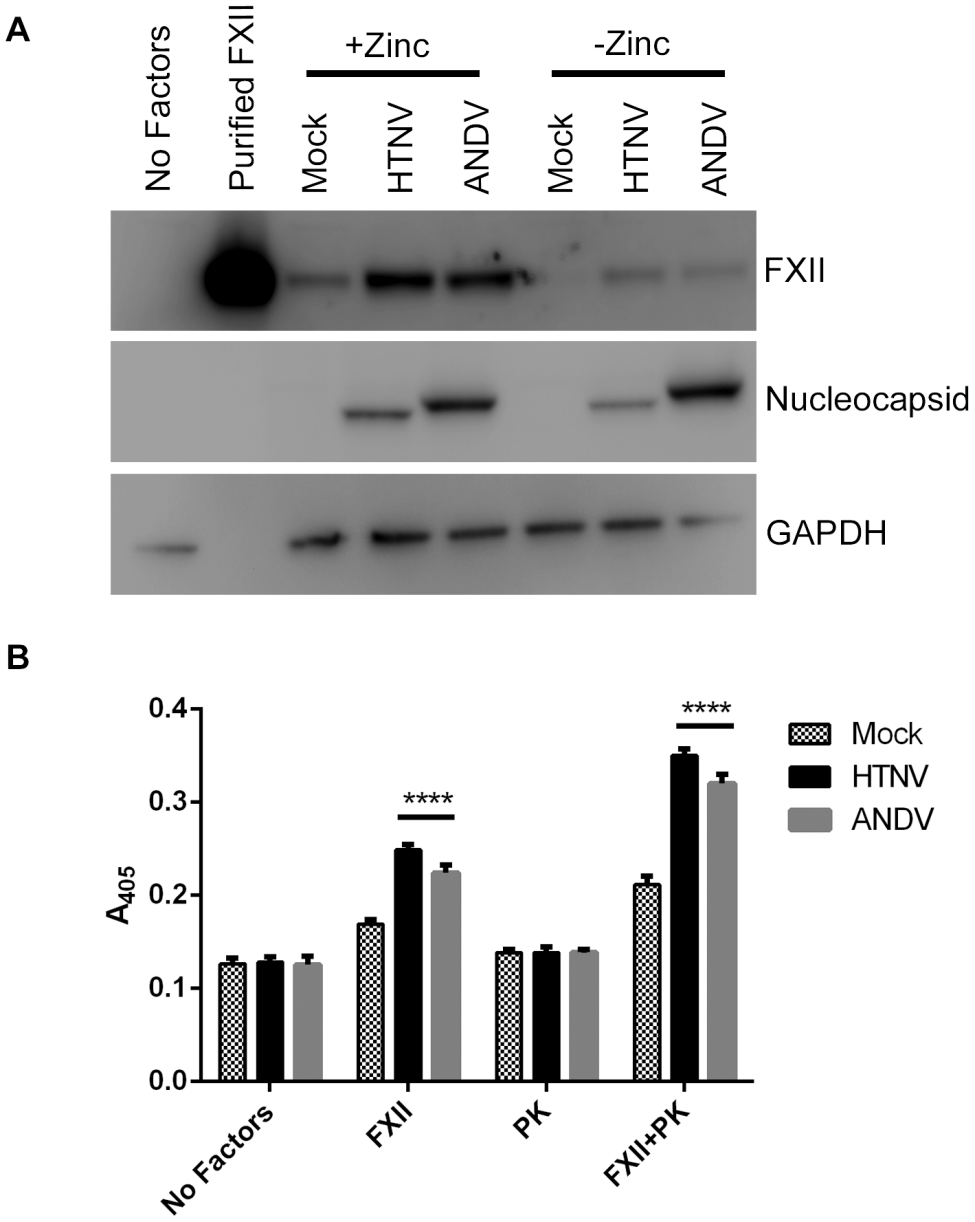
FXII binding and autoactivation on hantavirus-infected HUVEC. HUVEC were plated and mock-infected or infected with HTNV or ANDV for 4 days. (A) 100 nM of FXII in buffer was added to trypsinized cells in suspension and incubated for 1 h at 37°C. To examine FXII protein levels, cells were lysed and prepared for immunoblotting. FXII, hantavirus N, or GAPDH antibodies were used to detect protein. (B) To measure conversion of FXII to FXIIa, cells were washed and incubated with 25 nM FXII in the absence or presence of 25 nM PK and 0.8 mM S2302 for 2 h at 37°C. The liberation of paranitroanilide (pNA) from substrate (S2302) was measured at 405 nm. Data are represented as mean +/− SEM, n = 6. ****p≤0.0001.

Collectively, our data suggest that increased bradykinin liberation during hantavirus infection is dependent upon FXII. We hypothesized that FXII binding was increased on the surface of hantavirus-infected EC allowing for more activated enzyme in the cascade loop to amplify the reaction. We tested this by mock-infecting or infecting HUVEC with HTNV or ANDV. Cells were then trypsinized, washed, and left in suspension. FXII alone was added to samples in the presence and absence of zinc. Western blot analysis indicated that FXII levels were markedly increased on HTNV- and ANDV-infected HUVEC in the presence of zinc concentrations optimal for binding (Figure 8A). However, even in the absence of zinc, we were able to detect small amounts of FXII bound to HTNV- and ANDV-infected HUVEC (Figure 8A).

Since FXII alone is increased on the surface of hantavirus-infected EC, we also considered whether FXII could still autoactivate and if autoactivation was increased over levels of normal uninfected EC. Autoactivation of FXII to FXIIa requires 60–120 min. incubations on the surface of HUVEC [32]. HUVEC that were mock-infected or infected with HTNV or ANDV were incubated with FXII for 2 h. At 1 h, we detected no discernable difference in the generation of FXIIa as measured by the chromogenic substrate S2302 (data not shown). However, by 2 h enzyme activity was detectable in mock-, HTNV- , and ANDV-infected HUVEC suggesting that autoactivation was occurring (Figure 8B). Furthermore, FXIIa activity was significantly increased in HTNV- and ANDV-infected HUVEC when compared to mock (Figure 8B). This activity was further increased upon addition of PK, suggesting that FXIIa was present to convert PK to KAL (Figure 8B). In the absence of FXII, PK alone was not sufficient to hydrolyze S2302. Samples incubated in buffer alone revealed no increase in hydrolytic activity of S2302 between time 0 and 2 h, demonstrating that contaminating KAL was not present in our assay to account for the FXIIa formation (Figure 8B).

## Discussion

Here we show that KKS activation and BK liberation directly increases EC permeability *in vitro*, and we hypothesize that a similar mechanism contributes to the vascular leakage manifestations of hantavirus diseases *in vivo*. Previous studies suggested that hantavirus pathogenesis was related to degradation of VE-cadherin due to a hypersensitization of hantavirus-infected EC to VEGF [10], [11]. In another study, ANDV infection of cultured EC was found to result in increased levels of VEGF and VE-cadherin degradation, leading investigators to postulate that the stimulation of VEGF release by EC coupled with their sensitization to the effects of VEGF could result in the loss of EC barrier function [12]. Contrary to this hypothesis, our results obtained using an *in vitro* capillary blood vessel model comprised of both EC and vSMC and able to secrete VEGF, did not show increased VE-cadherin degradation or VEGF levels after infection with either the HFRS-causing virus HTNV, or the HPS-causing virus, ANDV. Our findings do not preclude the importance of VEGF and VE-cadherin in hantavirus permeability, but suggest that additional studies are needed to resolve these discrepancies in the different types of cell culture systems used.

As we showed, HTNV and ANDV can infect vSMC when cultured alone or in the *in vitro* capillary blood vessel model. Endothelial cells regulate barrier function and vascular tone in close collaboration with vSMC. Endothelial dysfunction as a result of injury or stress can trigger the production of EDHF, PGI_2_, and NO production [33]. These EC-derived factors act directly on vSMC to induce vasodilation. As a response to EC-derived factors, vSMC can upregulate the transcriptional activity of certain factors such as VEGF [34]. Testing has not been performed to determine if hantavirus-infected vSMC respond appropriately to EC-derived mediators. Our findings warrant additional studies on the role that hantavirus-infected vSMC play during pathogenesis. This will help to further dissect pathogenic mechanisms occurring locally in infected blood vessels.

The manifestations of HFRS and HPS; e.g., edema, hypotension, shock, and coagulation abnormalities, resemble clinical symptoms of bacterial diseases for which systemic KKS activation is thought to play an important role. Most notably, excessive KKS activation has been observed during meningococcal septic shock and streptococcal toxic shock syndrome [25], [35]. Both gram positive and gram negative bacteria have the ability to bind to one or more of the plasma components and directly cleave HK or proteolytically activate FXII and/or PK to indirectly release BK (reviewed in [36]). These types of enzymatic activities are not known to be associated with hantavirus proteins making it unlikely that the virus itself is directly activating FXII or PK through cleavage mechanisms. Endogenous activators of FXII have also been identified such as nucleic acids, misfolded proteins, and aggregates [37]–[39]. Since hantaviruses have no lytic effect on EC, it seems improbable that viral RNA or misfolded/aggregated proteins would be released from cells initiating FXII and PK activation. Tissue factor and anionic phospholipid is found incorporated into the virion of Herpes Simplex Virus-1 which allows for binding of plasma factors and initiation of coagulation cascades [40]. We have not observed any increase in activation of the KKS with HTNV alone. The more likely explanation is a hantavirus-directed effect that increases KKS activation.

In addition to bacterial diseases, a rare genetic disorder, HAE, has been associated with plasma factor activation dysfunctions. HAE is characterized by recurrent attacks of edema that can occur in the airway, extremities, face, and abdomen [41]. While the trigger for attacks is not well understood, the mechanism that produces edema has been very well characterized. Patients are deficient in C1 esterase inhibitor (C1-INH) which is important in suppressing FXIIa [41]. With reduced levels of C1-INH, patients experience uncontrolled FXII activation and subsequently elevated levels of BK resulting in edema formation [41]. Fortunately, there are three types of FDA approved drugs that target individual components of this pathway to treat disease. C1-INH concentrate (Berinart, Cinryze, Rhucin) can be administered to suppress FXIIa and KAL activity, Ecallantide (Kalbitor) is a specific inhibitor of KAL, and HOE 140 (Icatibant, Firazyr) targets the BKB2R to prevent binding of BK [42]. Although most of these drugs have not been tested in HFRS or HPS patients, recently, the BKB2R antagonist, icatibant, was given to a single patient with a severe case of Puumala virus-associated HFRS [26]. The patient improved and recovered after receiving one dose of icatibant, supporting the theory that BK might play a role in HFRS pathogenesis and suggesting that further controlled clinical studies of BK inhibitors are warranted.

Our *in vitro* studies demonstrate that increased cleavage of HK and permeability can be significantly inhibited in the presence of CTI, a specific inhibitor of FXIIa. Our data also shows that FXII binding is increased on the surface of hantavirus-infected EC. FXII activation can occur by two distinct mechanisms; proteolytic activation on the surface of EC by formed KAL or autoactivation through binding to biological surfaces. Our data suggests that both mechanisms could be important for increased KKS activation on the surface of hantavirus-infected EC. Increased binding of FXII to hantavirus-infected EC would facilitate increased formation of PK and subsequent liberation of BK, while binding of FXII alone could autoactivate to FXIIa and initiate the KKS. FXII binding is receptor specific on the surface of EC with a predominant complex utilized for preferential binding of FXII that includes urokinase plasminogen activator receptor (u-PAR) and cytokeratin 1 (CK1) [43]. FXII also autoactivates upon binding to transiently expressed globular heads of complement C1q receptor (gC1qR) [44]. Under physiologic conditions, binding of FXII is highly restricted by the concentration of zinc and HK present in the plasma [45]. *In vitro* studies have demonstrated that activated platelets can serve as a sufficient source of zinc for FXII activation [46]. *In vitro*, HTNV and ANDV are known to direct the adherence of platelets to the surface of EC, but there is yet no available data on whether platelets are activated. Future studies aim to identify changes occurring in hantavirus-infected EC that facilitates increased binding of FXII and whether platelets can contribute to binding and activation of FXII.

There are currently no data available on FXII activation during hantavirus infections; however, clinical studies have already been performed specifically examining KAL and vascular permeability in HFRS patients, providing evidence that KKS activation is also altered *in vivo*. In one clinical study, plasmas from 86 patients were collected over the course of disease and KAL levels were measured during each phase [47]. Additionally, patients were categorized based on the severity of disease; mild, moderately grave, and grave. The findings were dramatic with elevated levels of KAL detected in almost all of the patients. Even in mild cases, these levels were 8 times greater than the control values [47]. Moderately grave and grave cases demonstrated 10 and 12 fold increases in KAL activity providing a correlation to severity of disease [47]. In a separate study, a direct link between KAL and the permeability index was identified in 56 HFRS patients [48]. Rates of fluid and protein loss were directly associated with elevated levels of KAL. In this study, PK levels were also measured and were determined to be decreased except in patients with mild forms of disease [48]. Similar findings are commonly observed in other diseases, such as septic shock, where excessive activation of FXII and PK results in consumption and thus decreased total levels of factor [49]. There are currently no documented clinical or animal studies that have extensively examined FXII, PK, KAL, or levels of BK during the course HPS. However, examination of studies published from ANDV-infected hamsters and patients reveal indirect evidence of intrinsic pathway activity during disease including prolonged bleeding times, decreased fibrinogen, and fibrin deposition found in pathology [50]–[52]. The hamster model accurately reflects many aspects of HPS caused by ANDV infection in humans; it will be extremely interesting to examine these pathways in detail in the hamsters and the potential of drugs that impact them during the course of diseases.

**Figure 9:**
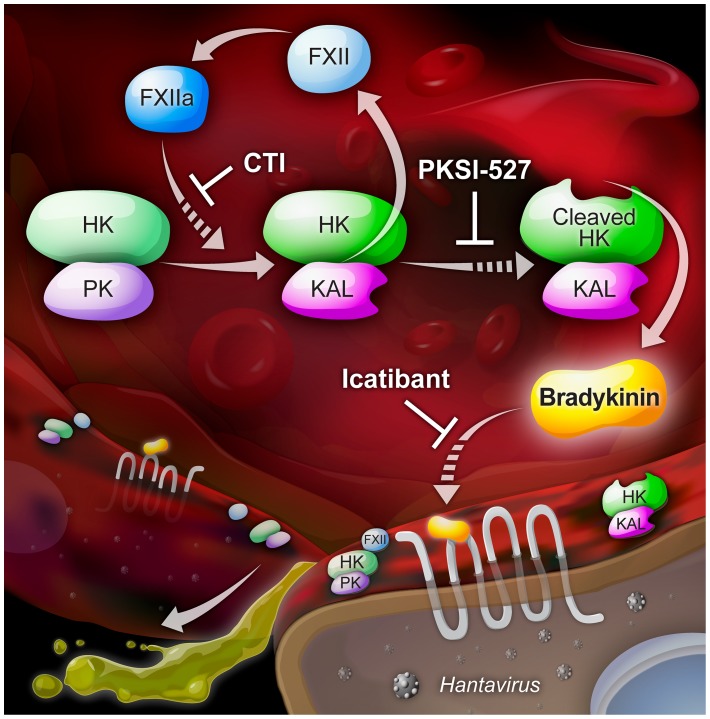
Kallikrein-kinin system and BK inhibitors. A schematic illustration of three inhibitors that impact BK-mediated increase in endothelial cell permeability. **CTI** (corn trypsin inhibitor) inhibits FXIIa; **PKSI-527** (plasma kallikrein specific inhibitor) inhibits KAL activity; **Icatibant** (HOE 140) is a peptidomimetic drug consisting of ten amino acids, which is a selective and specific antagonist of BKB2 receptors and is used in humans under orphan drug status for symptomatic treatment of hereditary angioedema.

To date, there are no therapeutics available to counteract acute symptoms of HFRS or HPS. If, as we conjecture, KKS activation and BK liberation are responsible for edema formation and hypotension during HFRS and HPS, then inhibitors targeting the effects of BK would presumably be effective as therapeutics for patients that are already presenting with symptoms. Figure 9 depicts three specific pathway junctions that we were able to target *in vitro* to block hantavirus-induced permeability. Our findings suggest the possibility of identifying multiple points of intervention using therapeutics. Some of these points can already be targeted by existing drugs used for other human diseases. Furthermore, identification of effective therapeutics that are already approved for use in humans could shorten time and expense to bring them to market. Lastly, although many hemorrhagic fever-causing viruses induce coagulation abnormalities, the underlying mechanisms of pathogenesis remain to be determined. It is possible that the pathways we have identified as contributing to hantavirus pathogenesis will also have broader implications for other viral hemorrhagic illnesses.

## Materials and Methods

### Cells, reagents, and virus

Human umbilical vein endothelial cells (HUVEC) were maintained in endothelial cell basal growth media (EBM-2) that was supplemented with 2% fetal bovine serum (FBS), 0.2 ml hydrocortisone, 2 ml hFGF-B, 0.5 ml VEGF, 0.5 ml R3-IGF-1, 0.5 ml ascorbic acid, 0.5 ml hEGF, 0.5 ml GA-1000, 0.5 ml heparin (Lonza). Human mesenchymal stem cells (hMSC) were maintained in mesenchymal stem cell basal growth media (MSCGM) supplemented with 10% mesenchymal cell growth serum (MCGS), 0.5 µg/ml of A/B, and 100 IU of Pen-Strep (Lonza). Human pulmonary artery smooth muscle cells (PaSMC) were maintained in smooth muscle basal media (SmBM-2) supplemented with 5% FBS, 0.5 ml heparin, 1 ml hFGF-B, 0.5 ml GA-1000, and 0.5 ml hEGF (Lonza). All primary cells were cultured in wells coated with 0.1% gelatin. *Cercopithecus aethiops* kidney cells, Vero C1008 clone E6 cells (Vero E6) were maintained in Eagle's Minimum Essential Medium (EMEM) supplemented with 10% FBS. All cells were incubated at 37°C, 5% CO_2_ for the indicated period of time.

HTNV strain 76–118 [53] and Andes virus strain Chile-9717869 (from Center for Disease Control, 4th pass of 808034, July 24th, 1998) were propagated in Vero E6 cells. Supernatants were harvested and clarified by centrifugation and titers of aliquots were determined by plaque assay.

### Enzymes, proteins, and biochemicals

Single chain HK, PK (specific activity of 28 PEU/mg), and FXII (specific activity of 29 U/mg) were purchased from Enzyme Research Laboratories. The substrate H-D-Pro-Phe-Arg-p-nitroanilide (S2302) was purchased from Diapharma (Franklin, OH). The antagonist, HOE 140 (icatibant) was used to block the BKB2R and Trandolapril to prevent cleavage of BK (Sigma Aldrich). PKSI-527 is a specific inhibitor of KAL activity (Enzo Life Sciences) and corn trypsin inhibitor (CTI) inhibits FXIIa (Haematologic Technologies Inc.).

### Immunohistochemistry and immunofluorescence

For immunohistochemistry and immunofluorescence of *in vitro* capillaries, HUVEC and PaSMC or hMSC were seeded, cultured and maintained for 5 days as previously described [27]. Samples were fixed with 10% formalin and permeabilized with 0.2% Triton X-100 in PBS. After blocking with 10% goat serum, samples were stained for markers to specifically detect HUVEC [vWF (Santa Cruz Biotechnology)], smooth muscle cells [SMA (Santa Cruz Biotechnology)], or Collagen 18A1 (Santa Cruz Biotechnology) overnight at 4°C. In HUVEC and PaSMC co-cultures, horseradish peroxidase (HRP) and alkaline phosphatase (AP) conjugated antibodies were used to visualize SMC and EC capillary tubes, respectively. DAB and vector blue (Vecta Labs) were used as chromogenic substrates. In HUVEC and hMSC co-cultures, Alexa Fluor 568 and 488 secondary antibodies were used to detect vWF and Collagen 18A1, respectively.

For hantavirus infection, *in vitro* capillaries or PaSMC alone were seeded on chamber slides and mock-infected or infected with HTNV or ANDV at a multiplicity of infection (MOI) of 2. For samples containing *in vitro* capillaries, the MOI was based on the initial number of HUVEC and PaSMC seeded in wells. After infection for 3 days, samples were fixed, permeabilized, and blocked as described for immunohistochemistry. Cells were stained with antibodies specific for vWF (Santa Cruz Biotechnologies), SMA (Santa Cruz Biotechnologies), or hantavirus nucleocapsid (N) (Abcam) overnight at 4°C. Alexa Fluor 568 and 488 secondary antibodies were used to detect vWF or SMA and hantavirus N, respectively. All coverslips were mounted on slides using Prolong Gold antifade reagent with DAPI (Life Technologies) and examined with a Leica DMI6000 B inverted microscope.

### Scanning electron microscopy

A co-culture of HUVEC and PaSMC or hMSC were grown (as described above to induce formation of capillary tubes) on 5×7 mm silicon chips (TedPella). Specimens were then fixed with 2.5% glutaraldehyde in 0.1 M sodium cacodylate buffer, post fixed with 1% osmium tetroxide in 0.1 M sodium cacodylate, and dehydrated with a graded ethanol series. The samples were critical point dried under carbon dioxide in a Bal-Tec model cpd 030 Drier (Balzers, Liechtenstein), mounted on aluminum studs, and sputter coated with 100 A of iridium in a model IBSe/SBT ion beam sputterer (South Bay Technologies) prior to viewing in a Quanta Field Emission Gun Scanning Electron Microscope (FEI) at 5.0 kV and a working distance of 10 mm.

### Western blots

Mock-infected, HTNV- or ANDV-infected cell lysates were prepared with NP-40 lysis buffer (150 mM NaCl, 50 mM Tris-HCl pH 8.0, 1% NP-40, and protease inhibitor cocktail), NuPAGE LDS sample buffer, and NuPAGE reducing agent (Life Technologies). Proteins were separated on 4 to 12% gradient polyacrylamide gels and transferred to polyvinylidene difluoride (PVDF) membranes. Blots were blocked with 5% non-fat milk in Tris-buffered saline (TBS) and subsequently probed overnight at 4°C with antibodies directed against HK (Abnova), FXII (Santa Cruz Biotechnology), PRCP (Santa Cruz Biotechnology), HSP90 (Santa Cruz Biotechnology), hantavirus N (ANDV NR-9673 BEI Resources), VE-cadherin (Santa Cruz Biotechnology), or GAPDH (Santa Cruz Biotechnology). Blots were then washed with TBS containing Tween 20 and probed with the appropriate species-specific HRP-conjugated secondary antibody. Blots were developed using a femto substrate detection system (Pierce Biotechnology) and signal captured using the G:Box (Stratagene).

### VEGF ELISA

Capillaries were seeded and mock-infected or infected with HTNV or ANDV at an MOI of 2 for 3 days. Supernatants were subsequently removed and measured using a VEGF ELISA kit according to the manufacturer's instructions (Life Technologies).

### Bradykinin enzyme immunoassay

Capillaries, HUVEC, or PaSMC were seeded and mock-infected or infected with HTNV or ANDV at an MOI of 2 for 3 days. To accurately measure BK levels in our supernatants, cells were pre-treated with 1 µM HOE 140 and 5 µM Trandolapril for 30 min. and throughout incubation with the plasma factors (FXII, PK, and HK) to prevent receptor binding and degradation of BK. FXII, PK, and HK were diluted to 50 nM each in HEPES (14.7 mM)-Tyrodes's buffer (Sigma Aldrich) containing 8 µM Zn^2+^, added to cells, and incubated for 1 h at 37°C. Supernatants were subsequently removed and measured using a BK EIA kit according to the manufacturer's instructions (Bachem).

### Cleavage of HK on HUVEC

HUVEC were seeded and mock-infected or infected with HTNV or ANDV at an MOI of 4 for 4 days. Media was removed and cells were washed with HEPES-Tyrode's buffer. HK was diluted to 50 nM in the presence of 1 µM Zn^2+^ HEPES-Tyrode's buffer, added to cells, and incubated at 37°C for 1 h. Cells were washed and incubated with FXII (50 nM) and PK (50 nM) diluted in 8 µM Zn^2+^ HEPES-Tyrode's buffer. In some experiments, CTI (1 µM) was added to inhibit FXIIa. At the end of 1 h incubation at 37°C, cells were washed and lysed in NP-40 lysis buffer and examined by western blotting. In certain experiments, only PK (50 nM) and HK (50 nM) were present in order to measure HK cleavage that is independent of FXII. These experiments were performed as described above except 1 µM Zn^2+^ concentrations were used throughout the incubation steps.

### FXII and PK activation on HUVEC

HUVEC were seeded and mock-infected or infected with HTNV or ANDV at an MOI of 4 for 4 days. Media was removed and cells were washed with HEPES-Tyrode's buffer. HK was diluted to 20 nM in the presence of 1 µM Zn^2+^ HEPES-Tyrode's buffer, added to cells, and incubated at 37°C for 1 h. Cells were washed and incubated with FXII (20 nM), PK (20 nM), and S2302 (0.8 mM) in the presence of 8 µM Zn^2+^ HEPES-Tyrode's buffer. Hydrolysis of the chromogenic substrate S2302 was measured by taking absorbance readings at 405 nm after 1 h incubation at 37°C.

### Electric cell-substrate impedance sensing

ECIS 8W10E+ arrays (Applied Biophysics) were coated with 10 µg/ml of poly-D-lysine (PDL) and washed with sterile water. EmbryoMax Ultrapure water with 0.1% gelatin (Millipore) was then added to each well and allowed to incubate for 1 h followed by washing with sterile water. After the final wash, 400 µl of EBM-2 media was added to each well. Arrays were then loaded into the ECIS 16-well array station and stabilized using ECIS software v1.2.92.0 (Applied Biophysics). HUVEC cells were mock-infected or infected with HTNV or ANDV and cultured for 4 days in flasks. After 4 days, cells were trypsinized and approximately 1×10^5^ cells were plated (per well) in the coated arrays. The array station was placed in a standard 37°C incubator with 5% CO_2_. ECIS software was used to run a multi-frequency time scan at 400, 4,000, 32,000, and 64,000 Hz to allow for collection of data related to resistance, impedance and capacitance. After approximately 18 h, data were visualized at 64,000 Hz and capacitance levels were uniformly below 10 nF (indicative of confluent monolayers). The instrument was paused and the array station moved to a Class II BSC. Media was removed from wells and replaced with 200 µl of phenol red free EBM-2 containing Zn^2+^ and inhibitors [CTI (1 µM), PKSI-527 (5 µM), or HOE 140 (1 µM)]. Cells were allowed to re-equilibrate at 37°C after which time an additional 200 µl of phenol red free EBM-2 containing Zn^2+^, FXII, PK, and HK (100 nM each) were added to wells in a Class II BSC. Data were collected real-time throughout the experiment and analyzed using ECIS Software.

### FXII binding to HUVEC

HUVEC were seeded and mock-infected or infected with HTNV or ANDV at an MOI of 4 for 4 days. Cells were trypsinized and washed with HEPES-Tyrode's buffer. FXII was added to cells in suspension at 100 nM in the presence or absence of 10 µM Zn^2+^ HEPES-Tyrode's buffer and incubated at 37°C for 1 h. Cells were washed and prepared as described for western blotting. To exclude the possibility that FXII was non-specifically binding to extracellular matrix proteins produced during culturing, cells were trypsinized and binding performed in suspension.

### FXII autoactivation on HUVEC

HUVEC were seeded and mock-infected or infected with HTNV or ANDV at an MOI of 4 for 4 days. Prior to the addition of plasma factors, HUVEC were placed in serum free media overnight. Cells were washed with HEPES-Tyrode's buffer and incubated with FXII (25 nM), PK (25 nM), or FXII and PK, and S2302 (0.8 mM) in the presence of 8 µM Zn^2+^ HEPES-Tyrode's buffer. Hydrolysis of the chromogenic substrate S2302 was measured by taking absorbance readings at 405 nm after 2 h incubation at 37°C.

### Statistics

ELISA and enzyme activity data were analyzed using a Student's *t* test to determine significant differences between mock and HTNV or mock and ANDV samples. *P* values<0.05 were considered significant.

### Accession numbers

The following GenBank accession numbers refer to the proteins mentioned in the text: FXII, AAM97932.1; PK, AAF79940.1; HK, AAB59551.1; CTI, CAA37998.1.
